# *GYRB* – POLYMERASE CHAIN REACTION AND HISTOPATHOLOGIC CHARACTERISTIC FIGURE POTENTIAL FOR DETERMINING DIAGNOSIS OF TUBERCULOUS LYMPHADENITIS

**DOI:** 10.21010/Ajidv17i2S.3

**Published:** 2023-08-01

**Authors:** HERLAMBANG Wahyu, MERTANIASIH Ni Made, SOEDARSONO Soedarsono, SANDHIKA Willy

**Affiliations:** 1 Master Program of Tropical Medicine, Faculty of Medicine, Universitas Airlangga, Surabaya, Indonesia; 2 Department of Medical Microbiology, Faculty of Medicine, Universitas Airlangga, Surabaya, Indonesia; 3 Department of Clinical Microbiology, Dr. Soetomo Academic Hospital, Surabaya, Indonesia; 4 Sub-pulmonology of Internal Medicine, Faculty of Medicine, Hang Tuah University, Surabaya, Indonesia; 5 Department of Anatomical Pathology, Faculty of Medicine, Universitas Airlangga, Surabaya, Indonesia; 6 Tuberculosis Laboratory, Institute of Tropical Disease, Universitas Airlangga, Surabaya, Indonesia

**Keywords:** EPTB, FNAB aspirate, *gyrB*, PCR, tuberculosis, tuberculous lymphadenitis

## Abstract

**Background:**

TB lymphadenitis is still a problem that needs serious treatment. In Indonesia, it was reported that 53% of TB cases were extrapulmonary tuberculosis, with the most cases being Lymphadenitis TB, 11.6%. In children, 43% of extrapulmonary tuberculosis cases are TB lymphadenitis. Diagnosis is quite difficult; a method of determining the diagnosis and appropriate comprehensive treatment is required in managing TB Lymphadenitis.

**Materials and Methods:**

In this study, 15 fine needle aspiration biopsy aspirate samples were subjected to molecular examination using the gyrB–polymerase chain reaction method and histopathological observations using the smear method with hematoxylin-eosin staining. Observation of preparations using a microscope with a magnification of 200x.

**Results:**

The histopathological characteristics of the fine needle aspiration biopsy aspirate showed positive results in 4 out of 15 samples, with epithelioid cells arranged in a characteristic granuloma structure, necrotic debris was visible, and cells joined together to form multinucleated giant cells as an inflammatory response to *Mycobacterium tuberculosis* complex infection. In this study, 6 out of 15 (40%) were detected to be positive in the diagnosis based on molecular detection using a specific target gene gyrB - polymerase chain reaction .

**Conclusion:**

Characteristic features on histopathological examination associated with gyrB - positive polymerase chain reaction on lymphadenitis *fine needle aspiration biopsy* aspirate samples can be used as a determinant diagnosis of tuberculous lymphadenitis.

## Introduction

Tuberculosis is still an infectious disease with high morbidity and mortality globally. Based on reports in 2021, it is estimated that around 10 million people are diagnosed with TB, with a mortality rate of 15% annually (WHO, 2021). Approximately 20% of TB cases are extrapulmonary TB cases (EPTB). EPTB can attack various organs in the body, but generally, 50% of cases are found in the lymph nodes (Baykan et al., 2022). In Indonesia, it was reported that 53% of TB cases were EPTB, with the highest number of tuberculous lymphadenitis (TBL) cases being reported at 11.6%. It was also reported that 45% of pediatric EPTB patients were diagnosed with tuberculous lymphadenitis (Soekotjo et al., 2019; Anggraini and Oktora, 2021)

EPTB can be a primary manifestation if the organ is the initial site of infection, generally caused by non-tuberculous mycobacteria (NTM). It can also be a secondary manifestation if it results from spreading bacteria from the main organ. Secondary TB manifestations are the most common mechanisms that occur. The spread of bacteria can be through the bloodstream or lymph vessels and then manifest in the lymph nodes, where inactive macrophages phagocytose the bacteria. The bacteria then replicate within the macrophages until the proteolytic enzymes and cytokines from the macrophages cause cell death and release of the bacteria. Monocytes and activated macrophages will form granulomas to limit bacterial infection, the mechanism can continue, and granulomas turn into lesions with necrosis in the center and the edges of fibrous tissue. TB lymphadenitis can also reactivate latent TB and can be caused by direct spread to the oropharyngeal mucosa in children (Rodriguez-Takeuchi et al., 2019; Gopalaswamy et al., 2021) .

Cervical lymphadenitis is the most common form of tuberculous lymphadenitis; however, it can also involve lymph vessels throughout the body. Cervical lymphadenitis is the most common manifestation in children with good immune systems. The clinical manifestations are not so clear that it is challenging to distinguish TB Lymphadenitis from mild inflammatory conditions or other infections. The clinical picture is generally in the form of dense enlarged lymph nodes, sometimes accompanied by abscesses, and can cause sinuses, although it is scarce. Generally, enlargement of the gland is unilateral, single, and located on the right side of the cervix with a size of between 3-6 cm, and in some cases, there are lesions in the lungs (Chang et al., 2013; Zimmermann et al., 2015; Arbind et al., 2016; Gautam et al., 2018).

Diagnostic confirmation of suspected TB lymphadenitis using FNAB aspirate tissue specimen examination or biopsy, microscopic methods for detecting acid-fast bacteria (AFB), histopathology, culture, and molecular examination using polymerase chain reaction (PCR) techniques, as well as GeneXpert MTB/RIF. PCR examination is a complementary examination for TB lymphadenitis besides histopathology. It has been applied to detect Mycobacterium DNA sequences in various examination materials, one of which comes from aspirate from Fine Needle Aspiration Biopsy (FNAB) (Chakravorty et al., 2005; Ligthelm et al., 2011; Coetzee et al., 2014).

In the treatment of TB Lymphadenitis, there are also difficulties. In a study, it was reported that there was a recurrence after treatment in TB Lymphadenitis patients after 28 months. As many as 3.8% of patients experienced a recurrence seen from the clinical picture, namely the presence of enlarged glands. The cause of recurrence after treatment is unknown, but examinations showed no drug resistance (Ko et al., 2019).

This study used the gyrB *Mycobacterium tuberculosis* complex (MTBC) gene as a primer based on its role in the metabolic process of MTBC bacterial cell replication. DNA gyrase in MTBC bacterial cells is a hetero tetramer (A2B2) molecule consisting of *gyrA* and *gyrB*. It plays a role in maintaining DNA topology, *gyrB*, in particular, initiates ATP hydrolysis (Kashyap et al., 2018). Using ATP as a cofactor, gyrase controls the topology of the DNA by adding or removing supercoiled DNA. Gyrase facilitates DNA unwinding at the replication fork by introducing negative supercoils (Petrella et al., 2019). This process is related to the active replication of MTBC, giving rise to inflammatory manifestations in patients, namely swollen lymph nodes. The *gyrB* gene has a specific and conserved region in MTBC as a target for DNA amplification in the PCR method in this study. In the active replication of MTBC in tissue specimens of FNAB Lymphadenitis TB, it can be assumed that high levels of the gyrB gene are detected. With the PCR method, it can have high sensitivity and specificity.

## Materials and Methods

### Sample collection

The research sample was obtained from FNAB Lymphadenitis aspirate in patients with suspected Lymphadenitis TB, at Dr. Soetomo Academic Hospital Surabaya, from August to November 2020. The samples were examined histopathologically and Xpert MTB/RIF.

### PCR assay

DNA extraction is conducted due to procedure guidelines in the DNeasy Blood & Tissue Kit [Qiagen, Jerman].

DNA amplification using the PCR method. A positive control using *M. tuberculosis* H37Rv. Identification of the MTBC group utilizing a pair of primers to amplify the target gyrB gene with the strand lengths presented in Table 1.

**Table 1:** Primer sequences used in the PCR process

Gene	Primer	Sequences (5’-3’)	Size (bp)
*gyrB*	MTUB F	TCGGACGCGTATGCGATATC	1020
	MTUB R	ACATACAGTTCGGACTTGCG	

The amplified product was analyzed using electrophoresis with agarose gel. The gel was then placed on a UV transilluminator and documented using a digital camera (Sinha et al., 2016).

### Histopathological examination

The examination was performed using the smear method with hematoxylin-eosin (HE) staining. Observation of preparations using an Olympus microscope with a magnification of 200x.

### Ethical Considerations

This study received ethical approval, with certificate number 1636/KEPK/XI/2019 dated 9 November 2019, from the Health Research Ethics Committee of Dr. Soetomo Academic Hospital Surabaya.

## Results

Total suspected Lymphadenitis TB were 39 samples from August 2020 to November 2020, and 8 positive Xpert MTB/RIF (20.5%). In this study, 15 samples were subjected to molecular and histopathological examinations.

### PCR assay of *gyrB* gene from FNAB aspirate of lymphadenitis

The aspirate from FNAB was subjected to molecular examination using the gyrB - PCR method using the target *Mycobacterium tuberculosis* gryB gene with a sequence length of 1020 bp. The results of this study indicate that of the 15 aspirate samples, 6 (40%) of them showed positive results.

**Figure 1:**
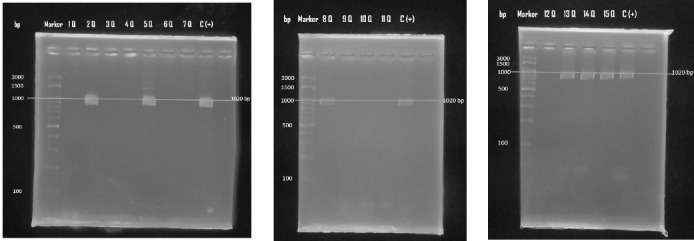
PCR examination results for detecting the gyrB gene for *M. tuberculosis* FNAB Lymphadenitis aspirate sample. The figure showed the 1020 bp band on the C(+) control of the M. tuberculosis H37Rv strain; note the positive band on samples 2, 5, 8, 13, 14, and 15.

From Figure 1, it is obvious that samples 2, 5, 8, 13, 14, and 15 showed the same 1020 bp DNA band (band) as the positive control band of *Mycobacterium tuberculosis* H37Rv. This stated that the six samples were positive for *Mycobacterium tuberculosis*.

### Histopathological examination analysis

**Figure 2:**
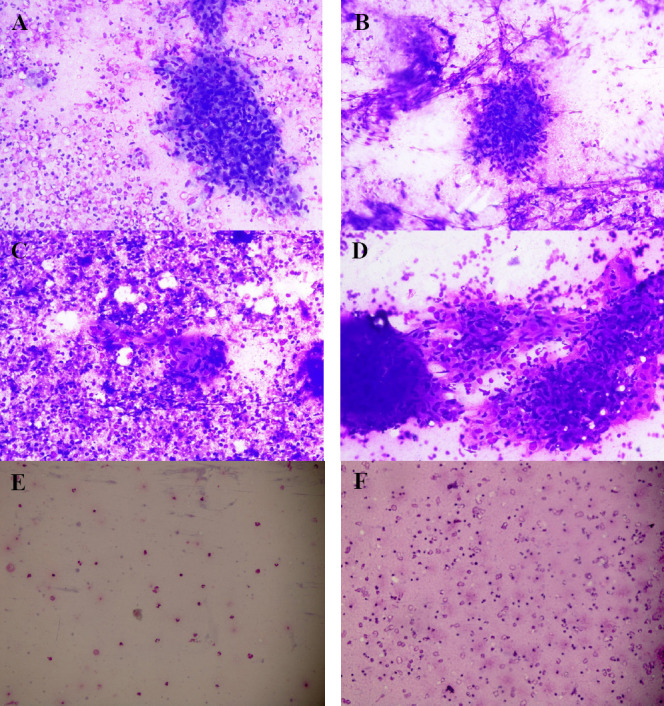
Histopathological examination of the FNAB aspirate showed positive results in images A-D. Images E and F show a negative result (200x Mag.)

The results of the histopathological examination of the molecularly positive samples are shown in Figure 2. Histopathologic characteristic features are positive for 4 of 15 samples (26.66%). In specimens that showed positive histopathological characteristics of tuberculosis, it was seen that the cell smear contained groups of epitheloid cells that formed granulomas against a background of lymphoid cells and a dense distribution of PMN inflammatory cells, as well as necrotic debris. Also seen are multinucleated giant cells, PMN cells, and amorphous debris. This suggests chronic granulomatous inflammation consistent with tuberculosis. In samples that showed a negative histopathological appearance, lymphocytes, PMNs and lymphoid cells of various maturities were distributed. No granuloma characteristic features were found, with the conclusion of chronic suppurative inflammation.

**Table 2:** FNAB aspirate specimens from patients with suspected TB lymphadenitis based on the results of histopathological examination, MTBC detection (gyrB-gene).

	*M. tuberculosis* (+)	*M. tuberculosis* (-)
**Histopatology (+)**	4	0
**Histopatology (-)**	2	9

## Discussion

In this study, 15 lymphadenitis FNAB aspirate samples were collected from patients, and six were positive for *Mycobacterium tuberculosis* PCR gyrB. In the other nine samples, MTBC was not found based on gyrB - PCR examination. In patients with negative gyrB - PCR test results, the lymphadenitis may be caused by NTM infection, other microbes, or malignancy (Willemse et al., 2018; Sarfaraz et al., 2018). Specific and conserved gyrB gene regions of MTBC detection can be stated as highly specific.

The paucibacillary nature of TB Lymphadenitis specimens makes diagnosis difficult and requires a combination of clinical, radiological, microbiological, and molecular examinations. Combined cytology characteristics (positive for epithelioid cell granuloma, multinucleated giant cells, and granuloma lesions with caseous necrosis) (Anggraini and Oktora, 2021; Djannah et al., 2022).

In FNAB, the aspirate material taken can be used for cytology testing, acid-fast bacilli (AFB) staining, culture, and molecular examination. FNAB cytology showed the formation of epithelioid granulomas and caseous necrosis. Such findings suggest a tubercle etiology, especially in developing countries with high TB. The sensitivity and specificity of FNAB cytology for the diagnosis of lymphadenitis were 88% and 96%. However, granulomas and caseous are rare in HIV-positive TB lymphadenitis due to impaired T-cell function. The combination of FNAB cytology and culture or rapid molecular test increases the diagnostic value of TB cervical lymphadenitis ( Kudu et al., 2020; Minnies et al., 2021).

The detection rate of *M. tuberculosis* in FNAB aspirates is relatively low by microbiological techniques. The positive value ranges from 15% - 47%, depending on the presence or absence of necrosis in patients with a history of TB. Cultures of the aspirate have been reported in some studies to be positive in 35% to 65%. In some cases, specimens were also found that were positive for TB on culture examination but negative on PCR examination, even after repeated trials. This is caused by very low levels of bacteria that cause false negatives on molecular analysis (Gautam et al., 2018; Singh, 2000).

In general, the concentration of DNA from the aspirate sample is low due to its paucibacillary nature. Inaccurate sampling techniques can also cause low DNA concentrations. The FNAB performed may need to be more precise on the granuloma so that bacteria are not found or only in very low numbers. This problem can be solved by using computed tomography (CT) guidance. The positivity rate of using CT guiding on FNAB examination was 95.4% in lymphadenitis patients (Kiral et al., 2015). In this case, CT guiding must be carried out as a mandatory procedure to improve the quality of diagnosis of TB Lymphadenitis patients.

## Conclusion

In this study, six samples (40%) positive of 15 FNAB samples of suspected Lymphadenitis TB patients were detected in the diagnostic based on molecular detection using a specific and conserved gene target, gyrB – PCR. Characteristic features of the histopathological examination in conjunction with positive gyrB - PCR in the FNAB of the Lymphadenitis aspirate sample can be used to determine Lymphadenitis TB.
